# IFN-gamma-induced PD-L1 expression in melanoma depends on p53 expression

**DOI:** 10.1186/s13046-019-1403-9

**Published:** 2019-09-11

**Authors:** Alexander Thiem, Sonja Hesbacher, Hermann Kneitz, Teresa di Primio, Markus V. Heppt, Heike M. Hermanns, Matthias Goebeler, Svenja Meierjohann, Roland Houben, David Schrama

**Affiliations:** 1 Department of Dermatology, University Hospital Würzburg, Würzburg, Germany; 2 Comprehensive Cancer Center Mainfranken, University Hospital Würzburg, Würzburg, Germany; 3 Department of Dermatology, University Medical Center Rostock, Rostock, Germany; 4 Department of Dermatology, University Hospital of Munich (LMU), Munich, Germany; 5 Medical Clinic II, Division of Hepatology, University Hospital Würzburg, Würzburg, Germany; 6 Department of Physiological Chemistry, Biocenter, University of Würzburg, Würzburg, Germany

**Keywords:** Melanoma, PD-L1, CD274, p53, TP53, JAK2

## Abstract

**Background:**

Immune checkpoint inhibition and in particular anti-PD-1 immunotherapy have revolutionized the treatment of advanced melanoma. In this regard, higher tumoral PD-L1 protein (gene name: *CD274*) expression is associated with better clinical response and increased survival to anti-PD-1 therapy**.** Moreover, there is increasing evidence that tumor suppressor proteins are involved in immune regulation and are capable of modulating the expression of immune checkpoint proteins. Here, we determined the role of p53 protein (gene name: *TP53*) in the regulation of PD-L1 expression in melanoma.

**Methods:**

We analyzed publicly available mRNA and protein expression data from the cancer genome/proteome atlas and performed immunohistochemistry on tumors with known *TP53* status. Constitutive and IFN-ɣ-induced PD-L1 expression upon p53 knockdown in wildtype, *TP53*-mutated or JAK2-overexpressing melanoma cells or in cells, in which p53 was rendered transcriptionally inactive by CRISPR/Cas9, was determined by immunoblot or flow cytometry. Similarly, PD-L1 expression was investigated after overexpression of a transcriptionally-impaired p53 (L22Q, W23S) in *TP*53-wt or a *TP53*-knockout melanoma cell line. Immunoblot was applied to analyze the IFN-ɣ signaling pathway.

**Results:**

For *TP53*-mutated tumors, an increased *CD274* mRNA expression and a higher frequency of PD-L1 positivity was observed. Interestingly, positive correlations of *IFNG* mRNA and PD-L1 protein in both *TP53*-wt and -mutated samples and of p53 and PD-L1 protein suggest a non-transcriptional mode of action of p53. Indeed, cell line experiments revealed a diminished IFN-ɣ-induced PD-L1 expression upon p53 knockdown in both wildtype and *TP53*-mutated melanoma cells, which was not the case when p53 wildtype protein was rendered transcriptionally inactive or by ectopic expression of p53^L22Q,W23S^, a transcriptionally-impaired variant, in *TP53*-wt cells. Accordingly, expression of p53^L22Q,W23S^ in a *TP53*-knockout melanoma cell line boosted IFN-ɣ-induced PD-L1 expression. The impaired PD-L1-inducibility after p53 knockdown was associated with a reduced JAK2 expression in the cells and was almost abrogated by JAK2 overexpression.

**Conclusions:**

While having only a small impact on basal PD-L1 expression, both wildtype and mutated p53 play an important positive role for IFN-ɣ-induced PD-L1 expression in melanoma cells by supporting JAK2 expression. Future studies should address, whether p53 expression levels might influence response to anti-PD-1 immunotherapy.

## Background

Antibodies directed against the cytotoxic T-lymphocyte-associated antigen 4 (CTLA-4) receptor or programmed cell death-1 (PD-1) receptor have revolutionized the systemic therapy of advanced melanoma [1]. Indeed, with these immunotherapeutic approaches durable responses in the treatment of metastatic melanoma have been achieved for the first time [2–4]. In the first-line setting, response rates to CTLA-4 or PD-1 blockade range between 10 and 19% or 40–45%, respectively [5, 6]. Moreover, when both antibodies are combined, response rates increase up to 61% [5]. Since these therapies, especially in case of combination, may be accompanied by major, possibly even life-threatening side effects, much effort is being spent on identifying predictive biomarkers. So far, the most commonly used predictor of therapeutic response to PD-1 blockade is the expression of programmed-death-ligand 1 (PD-L1), a ligand of PD-1, on tumor cells. PD-L1 (also denoted as B7-H1) is encoded by the *CD274* gene and is one of the two known ligands for PD-1, a costimulatory molecule that negatively regulates T-cell immune responses [7]. Notably, PD-L1 positivity (> 5% or > 1% of tumor cells positive for PD-L1 staining) is associated with a better overall response rate (ORR), progression free survival (PFS) and overall survival (OS) to anti-PD-1 immunotherapies [3, 4, 6, 8–10].

PD-L1 expression is inducible in many cell types, and increased expression has been observed in several tumor entities including melanoma, non-small cell lung cancer (NSCLC) and renal cell carcinoma. The interaction of cancer cell PD-L1 with PD-1 on cytotoxic T-lymphocytes (CTL) results in diminished T-cell killing [11, 12]. Possible mechanisms include suppressed T-cell proliferation and T-cell activation [13], induction of T-cell apoptosis [14] and also differentiation of CD4+ T-cells into FOXP3+ regulatory T-cells [15].

Various mechanisms have already been described that regulate PD-L1 expression in an often cell type-dependent manner [16]. Upregulation in tumor cells can be due to constitutively active oncogenic signaling pathways (referred to as *innate immune resistance*); although for melanoma cell lines PD-L1 expression levels were variable and independent from any driver mutation in MAPK or PI3K pathway [11, 17]. In addition, PD-L1 expression may occur in response to tumor-targeting immune cells that release interferon (IFN)-ɣ upon recognition of their cognate antigen expressed by cancer cells. PD-L1 expression on cancer cells subsequently inhibits PD-1-positive T-cells, a process known as *adaptive immune resistance* [11, 12]. IFN-ɣ signals through the canonical type II interferon receptor pathway [12, 18]. When IFN-ɣ binds to the IFN-ɣ receptor, JAK2 is activated by autophosphorylation of two tyrosine residues (Tyr 1007/Tyr 1008), and then transphosphorylates JAK1 on Tyr1022/Tyr1023. This leads to phosphorylation of tyrosine 440 in the IFN-ɣ receptor 1 by JAK1, which serves as recruitment site for STAT1 then allowing its phosphorylation on Y701 by most likely JAK2 [18, 19]. Subsequently, activated STAT1 dimers accumulate in the nucleus and act as transcription factors binding to the GAS (interferon-gamma activated site) elements of IFN-ɣ-inducible genes. The most important of these genes is interferon regulatory factor 1 (IRF1), which itself acts as a transcription factor during its ligation to IRF1-binding site-containing promoters like the PD-L1 promoter [18, 19]. Further transcriptional factors involved in PD-L1 regulation in melanoma include MYC, hypoxia-inducible factor-1α and 2α (HIF-1α/2α), STAT3 and NF-κB [16].

Post-transcriptionally, PD-L1 expression can be negatively regulated by various microRNAs (miRNAs, miR) such as miR-17-5p, miR-138-5p, miR-197, miR-200, miR-424, miR-513 and miR-570 [16, 20–26]. In addition, Cortez et al. recently demonstrated that p53 transcriptional activity leads to elevated miR-34a expression, which in turn reduced PD-L1 expression in NSCLC [27].

The main function of the tumor suppressor p53 is the regulation of cell proliferation and the induction of death in cells, which harbor genomic abnormalities [28, 29]. The molecular structure of p53 protein encompasses six domains (given residues are based on [30], but vary between studies): the transactivation domain (TAD) (residues 1–67), which can further be subdivided in two TADs; the proline-rich region (residues 68–98); the DNA-binding domain (DBD, residues 98–292); the hinge domain (HD, residues 293–325); the oligomerization domain (OD, residues 326–353); and the carboxy-terminal regulatory domain (CTD, residues 353–393). Most *TP53* mutations occur in the DBD, and by impaired binding to target gene DNA its tumor suppressor ability is often reduced [28]. In addition of losing its tumor-suppressive properties, stabilized mutant p53 may gain novel functions (referred to as gain-of-function, GOF) [28, 29]. Those GOF are able to promote tumor progression or produce resistance to antitumor therapies.

Since in melanoma *TP53* is frequently wildtype, we hypothesized that p53 might play a key role in repressing PD-L1 expression in melanoma and therefore investigated the role of p53 in PD-L1 regulation in melanoma.

Indeed, by conducting knockdown experiments of p53 in melanoma cell lines as well as immunohistochemistry of PD-L1 in melanoma tissue and analyzing the cancer genome atlas (TCGA) database, we found evidence for p53 being involved in the regulation of PD-L1 expression. Furthermore, we observed that induction of PD-L1 by IFN-ɣ is reduced after p53 knockdown. This is partly due to a reduction of JAK2, since ectopic JAK2 expression can largely rescue the effect of p53 knockdown on IFN-ɣ induced PD-L1 expression.

## Material and methods

### Cell lines, reagents and treatment regimens

We studied well-characterized melanoma cells from the NCI-60 panel that were *TP53*-wildtype (wt), i.e. LOX-IMVI, M19-MEL, MALME-3 M, SK-MEL-5, UACC-62, UACC-257, or *TP53*-mutated, i.e. M14, MDA-MB-435, SK-MEL-2 and SK-MEL-28. Furthermore, we used self-established *TP53*-mutated melanoma cell lines MS149 (p53 Q199*) and MS186 (p53 E154K). The two NCI-60 *TP53*-wt NSCLC cell lines A549 and H460 served as controls. All melanoma NCI-60 cell lines were obtained from ATCC. The 1205Lu melanoma cell line was originally obtained from the Wistar Institute, and the *TP53*-knockout variant was generated by Prof. Veit Hornung, Bonn/Munich, Germany, and kindly provided by co-author Markus Heppt, Munich.

Cell lines were cultured in RPMI-1640 with 10% fetal bovine serum, 100 U/ml penicillin and 0.1 mg/ml streptomycin (all from Sigma-Aldrich, Darmstadt, Germany) at 37 °C with 5% CO_2_. For IFN-ɣ stimulation, we used a concentration of 200 IU/ml (ImmunoTools, Friesoythe, Germany) for up to 48 h. Cell lines were regularly tested for mycoplasma contamination.

### Lentiviral transduction and generation of small hairpin RNAs

For knockdown experiments we transduced cells with an antibiotic-selectable (zeocin or blasticidin), doxycycline (Dox)-inducible lentiviral small hairpin (sh) RNA vector. These vectors are based on a previously described system and the sequence is deposited (Accession number MH749464) [31]. The *TP53* targeting sequences used were 5′-GAC TCC AGT GGT AAT CTA CT-3′ or 5′-CAC CAT CCA CTA CAA CTA CAT-3′ (in the confirmation experiments). A scrambled shRNA sequence (scr) served as control. Lentiviral transduction was performed as described before [32] and knockdown efficacy was determined by immunoblot. Cells containing doxycycline-inducible p53 or scr shRNA were incubated with doxycyline (1 μg/ml) for 6 days.

### CRISPR/Cas mediated p53 inactivation

CRISPR/Cas technology was used to render wt p53 transcriptionally inactive by introducing deletions in the DBD/HD region. To achieve p53 inactivation, cells were transducted with a lentiviral system consisting of Dox-inducible Cas9 (pCW-Cas9 was a gift from Eric Lander & David Sabatini, Addgene plasmid # 50661) and lentiguide for gRNA expression (lentiGuide-Puro was a gift from Feng Zhang, Addgene plasmid # 52963). Used guides were 5′-CAT GTG TAA CAG TTC CTG CA-3′ (exon 7) and 5′-GTG AAA TAT TCT CCA TCC AG-3′ (exon 9) for LOX-IMVI and 5′-AGA TTA CCA CTA CTC AGG AT-3′ (exon 8) and 5′-GGA GAG GAG CTG GTG TTG TT-3′ (exon 9) for UACC-62, respectively. Exon 7 and 8 encode amino acids belonging to the DBD, while exon 9 contributes amino acids to the HD. These two cell lines contained a GFP based p53 reporter construct to measure p53 activity, as previously described [33].

### Ectopic JAK2 and p53^L22Q,W23S^ expression

JAK2 expression plasmid (pUNO1-hJAK2, Invivogen, San Diego, CA, USA) was kindly provided by Prof. Annette Paschen (Dept. of Dermatology, University Hospital Essen, Germany) and cloned into a pCDH-based lentiviral vector. Two melanoma cell lines (M19-MEL, UACC-62), already containing the zeocin-selectable, Dox-inducible p53 or scr shRNA, were transduced with this vector. JAK2 expression was confirmed by immunoblotting.

For inducible ectopic expression of an p53 variant severely compromised for transactivation we mutagenized a pCW-based vector encoding a Dox-inducible flag-HA-tagged *TP*53-wt at positions 22 and 23 to generate p53^L22Q,W23S^ using the QuickChange Lightning Site-directed Mutagenesis kit (Agilent, Frankfurt, Germany) [34]. Two *TP*53-wt melanoma cell lines (M19-MEL, UACC-62) as well as a *TP53*-knockout melanoma cell line 1205Lu were transduced with this lentiviral vector.

### Immunoblotting

Total cellular proteins were extracted at 4 °C using erythrocytes lysis buffer (ELB) containing protease inhibitors (Roche, Basel, Switzerland). Proteins (10–20 μg) were resolved on 8–12% SDS–polyacrylamide gels and transferred to Amersham™ Protran™ Premium 0.45 μm NC (GE Health Care Europe, Freiburg, Germany).

Immunoblots were probed with antibodies against PD-L1 (E1L3N), JAK1 (6G4), phospho-JAK1 (Tyr1022/1023; D7N4Z), JAK2 (D2E12), phospo-JAK2 (Tyr1008; D4A8), phospho-STAT1 (Tyr701; 58D6; all by Cell Signaling Technology, Boston, MA, USA), STAT1 (10C4B40), phospho-STAT1 (S727; A15158B), IRF-1 (13H3A44; all by BioLegend, San Diego, CA, USA), monoclonal p53 (DO-1; Santa Cruz Biotechnology, Dallas, TX, USA) or polyclonal p53 (#9282; Cell Signaling Technology). ß-tubulin (TUB2.1) or ß-actin (AC-15; both Sigma-Aldrich) served as loading control.

### Flow cytometry

Adherent cells were detached using 0.02% ethylenediaminetetraacetic acid (EDTA) in phosphate buffered saline (PBS). After washing them with 0.1% bovine serum albumin (BSA; all from Sigma-Aldrich) in PBS, cells were incubated with PD-L1 APC antibody (29E.2A3, 1:20; BioLegend) or with HLA-ABC (MHC class I) APC antibody (W6/32; 1:50; ImmunoTools) for 20 min on ice. After washing twice with 0.1% BSA in PBS cells were analyzed with the BD FACS Canto. Non-viable cells were excluded using 7-Amino Actinomycin D (7-AAD; BD Biosciences, Franklin Lakes, NJ, USA).

### Immunohistochemistry

All analyzed samples were collected from patients who received treatment at the Department of Dermatology at the University Hospital Würzburg between November 2014 and July 2016. Written informed consent was obtained from each patient.

*TP53-*mutation status was determined by next generation sequencing (for detailed information on library preparation and sequencing, please refer to Appenzeller et al. [35]).

Four μm-sections of paraffin-embedded primary and metastatic tumors were dried at 75 °C for 20 min and then treated twice with xylol for 5 min at room temperature. Subsequently, sections were washed twice with absolute ethanol and once with 70% ethanol followed by one rinse with bi-distilled water. For antigen retrieval, sections were incubated with Tris/EDTA-buffer pH 9.0 for 40 min at 90 °C and then cooled down for 20 min. After washing in TBS buffer for 5 min, slides were incubated with monoclonal antibody against PD-L1 (E1L3N, CST, 1:200) for 40 min. Washing in TBS buffer for 5 min was followed by incubation with secondary antibodies (REAL Biotinylated Secondary Antibodies (AB2), Dako) for 30 min. Samples were briefly washed with TBS buffer again, then incubated in peroxidase-blocking solution for 5 min, streptavidin peroxidase for 25 min, CHROM AEC/H2O2 substrate solution for 15 min, automation hematoxylin histological staining reagent (all from Dako) for 5 min, and cleansed with bi-distilled water. Between every incubation step, slides were washed with TBS buffer.

### Analysis of the cancer genome atlas (TCGA)

TCGA data (http://cancergenome.nih.gov/) were retrieved and analyzed by software *R* with different packages [36]. In this regard, RNAseq and miRNA data for the cases of patients diagnosed with cutaneous melanoma, were downloaded and prepared with the package “TCGAbiolinks” [37]. Mutational data of *TP53* (i.e. missense, truncated, frameshift, splice mutations and homozygous deletions) was downloaded from cBioPortal and prepared by the package “maftools” [38]. Reverse phase protein array (RPPA) was obtained from the cancer proteome atlas (TCPA) [39]. For mRNA expression the transcripts per million (TPM) values, for miRNA the reads per million (RPM) and for the RPPA data the level 4 data generated by replicate-based method processing was extracted. Each data set was log2-transformed after adding the value 1 for TPM and RPM and 5.99 for RPPA. The mRNA and miRNA data were available for 447, the mutational data for 347 and the RPPA for 354 individual cases. All data were available for a cohort of 262 cases. For correlation analysis, the mRNA and miRNA datasets extracted from the RNAseq data available from the TCGA database was first restricted to those genes, which had at least 1 TPM (for correlation with genes) or at least 1 RPM (for correlation with miRNA) in 20% of the 447 cases using the R-package edgeR [40]. Subsequently, *CD274* mRNA expression was correlated to either the expression of the remaining genes or miRNA using the built in spearman correlation and loop function of R.

### Real-time PCR for quantification of *TP53*, *CD274* and *JAK2* mRNA expression

mRNA isolation, cDNA transcription and RT-qPCR with RPLP0 as endogeneous control was performed as described before [41]. Cells were treated for 6 days with doxycycline in the absence or presence of IFN-ɣ for the last 2 days. Primers used to detect the expression of the respective gene of interest by SYBR green assay were the following: *TP53*_fw: GAG GTT GGC TCT GAC TGT ACC; *TP53*_rv: TCC GTC CCA GTA GAT TAC CAC; *JAK2*_fw: CAG GCA ACA GGA ACA AGA TG; *JAK2*_rv: CCA TTC CCA TGC AGA GTC TT; *CD274*_fw: CAT CTT ATT ATG CCT TGG TGT AGC A; *CD274*_rv: GGA TTA CGT CTC CTC CAA ATG TG; RPLP0_fw: CCA TCA GCA CCA CAG CCT TC; RPLP0_rv: GGC GAC CTG GAA GTC CAA CT. Relative expression was calculated by the ΔΔCq method with the cells treated without doxycycline and IFN-ɣ serving as calibrator [42].

### Statistics

Statistical analysis was performed with *R*. Graphs were prepared with “ggplot2” [43]. Expression data between groups were compared by the Wilcoxon–Mann–Whitney test. Relations between two genes were calculated by linear regression. Correlations of genes, miRNA and protein expressions was calculated by Spearman correlation on filtered data (> 1 TPM or > 0 RPM, respectively, in at least 20% of cases). The factors with the best correlation estimate values were then depicted as heatmap generated with the package “ComplexHeatmap” [44] using Spearman as clustering distance and ward. D as clustering method for the factors. A *p* value < 0.05 was regarded significant. Gene enrichment analysis was performed using PANTHER overrepresentation test (version 13.1, released 2018.02.03) and Fisher test statististics [45, 46]. FCM data and relative mRNA expression (log2 transformed) were depicted and analyzed by Prism 7 (Graphpad) using a paired T-test.

## Results

### *TP53*-mutated melanoma present higher *CD274* mRNA expression levels

To allow for an easier discrimination between mRNA and protein in our manuscript, we refer to the official gene names for mRNA (*TP53* and *CD274*) and to the widely-used molecule names p53 and PD-L1 (CD274) for protein expression.

**Fig. 1:**
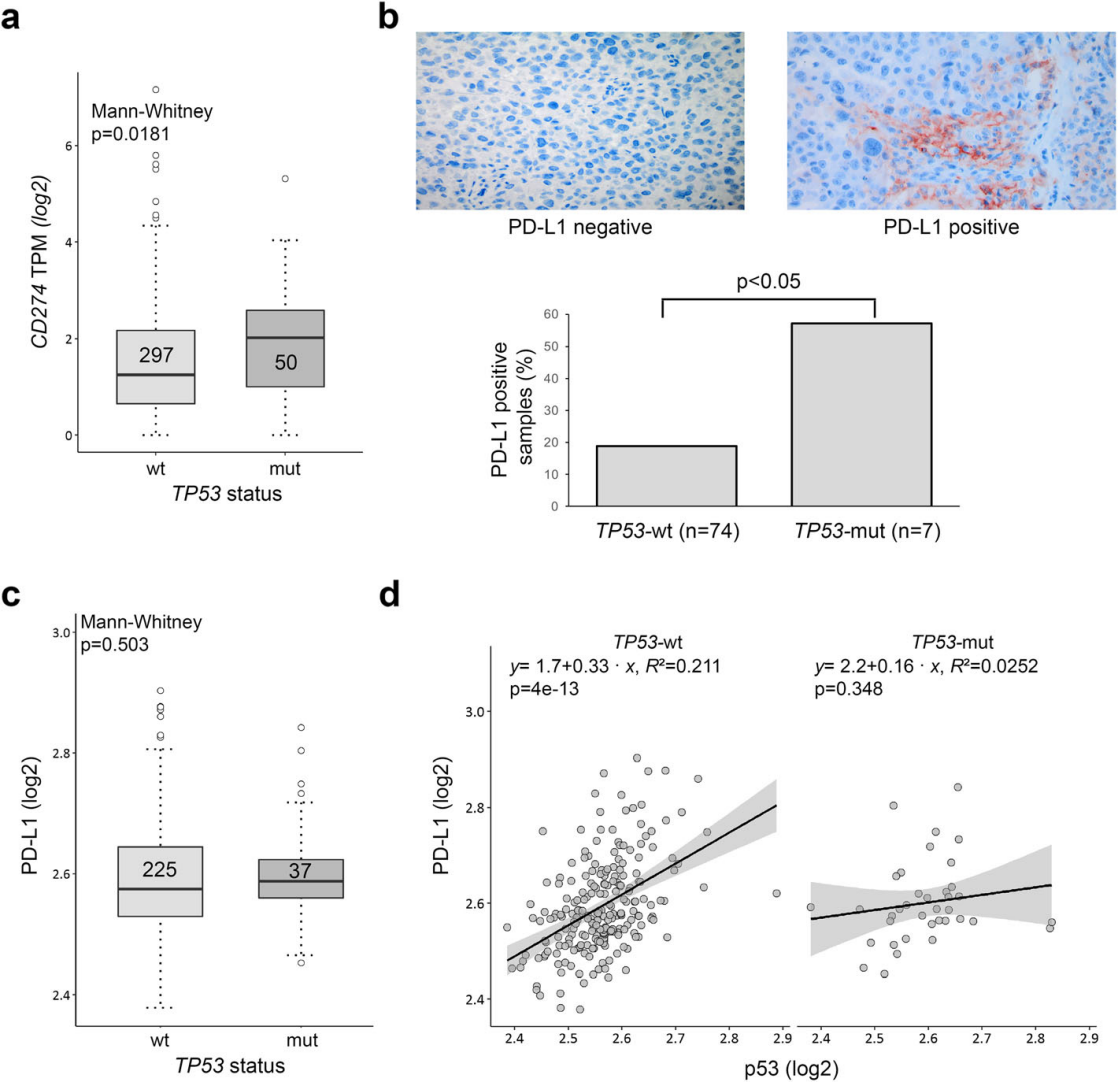
*TP53-*mutated melanoma show higher *CD274* mRNA expression levels, and are immunohistochemically more often PD-L1 positive. **a** Analysis of TCGA melanoma samples (*n* = 347) for differences in *CD274* mRNA expression. Wilcoxon-Mann-Whitney test was used to compare *CD274* expression between *TP53*-wt and -mutant samples. **b** PD-L1 immunohistochemistry of primary and metastatic melanoma samples (*n* = 81). Next-generation sequencing of the *TP53* gene was performed previously. Samples harboring more than > 1% PD-L1 positive melanoma cells were regarded as PD-L1-positive. A PD-L1 negative, and a positive metastasis are depicted. Magnification, each 400x (**c**, **d**) Samples (*n* = 262) were stratified by *TP53-*mutation status and publicly available PD-L1 expression determined by reverse phase protein array was compared with the Wilcoxon-Mann-Whitney test (**c**) and by linear regression association analysis with p53 (**d**). mRNA and protein data are presented on a logarithmic scale. *p* < 0.05 is regarded as statistically significant. TPM, transcripts per million; wt, wildtype; mut, mutation

Cortez et al. [27] analyzed NSCLC data from TCGA and reported significantly higher *CD274* mRNA levels in *TP53*-mutated NSCLC than in wt counterparts. Furthermore, they revealed a statistically inverse correlation between *TP53* and *CD274* mRNA for the total cohort of lung adenocarcinoma cases. To test a possible correlation between *TP53* and *CD274* in melanoma, we analyzed TCGA skin cutaneous melanoma (SKCM) data. To this end, also for melanoma *CD274* mRNA levels were significantly higher in *TP53*-mutated than in *TP53*-wt samples (Fig. 1a; *p* = 0.0181; Mann-Whitney).

In a next step, we performed immunohistochemistry for PD-L1 on 81 primary and metastatic melanoma samples with known *TP53-*mutation status, which has been determined by next generation sequencing before [34]. Regarding PD-L1 staining, it is common practice to use a threshold of either 1% or 5% of stained tumor cells to classify tumor samples [3, 4, 6, 8, 9]. Here we scored samples with > 1% stained tumor cells as PD-L1-positive. Noteworthy, 4/7 (57%) of *TP53*-mutated samples but only 14/74 (19%) of *TP53*-wt samples (*p* = 0.0401) were PD-L1-positive (Fig. 1b; for clinical information on this patient cohort see Additional file 1: Figure S1a).

In order to expand our investigation on PD-L1 expression in melanoma, we analyzed data obtained from the cancer proteome atlas (TCPA) project, which collects results from reverse phase protein array (RPPA) for different entities including melanoma [39]. These data did not reveal a statistically significant difference in overall PD-L1 expression levels between *TP53*-wt or -mutated melanoma samples (Fig. 1c). We did, however, detect a positive correlation between PD-L1 and p53 protein expression, which reached statistically significance only for the *TP53*-wt cohort (*p* = 4 × 10^− 13^; *R*^*2*^ = 0.211; Fig. 1d).

### miR-34a is not the key regulator of PD-L1 in TCGA skin cutaneous melanoma

For NSCLC, p53-driven miR-34a expression was demonstrated to be a key regulator of PD-L1 expression [27]. Since at least at the mRNA level the observed dependency of *CD274* on the *TP53*-mutational status was similar for melanoma as for NSCLC, we analyzed the melanoma TCGA data for miRNA and *CD274* expression focusing first on miR-34a. Again, similar as for NSCLC, miR-34a expression was significantly higher in *TP53*-wt than in *TP53*-mutated tumors (*p* = 0.0181; Additional file 2: Figure S2a), even with a small, but significant negative association of *TP53* mRNA and miR-34a expression (*p* = 0.00057; *R*^*2*^ = 0.0395; data not shown). However, no correlations between miR-34a and *CD274* mRNA (data not shown) nor between miR-34a and PD-L1 protein (Additional file 2: Figure S2b) were detected. Accordingly, neither miR-34a nor most of the other known miR negatively affecting PD-L1 were among the top 24 miRs correlating with *CD274* mRNA expression (Additional file 2: Figure S2c). The best correlation was observed for *CD274* mRNA and miR-7702 (Additional file 2: Figure S2d; *p* = 2 × 10^− 16^; *R*^*2*^ = 0.353), whose expression, however, was not significantly different between *TP53-*wt and -mutated tumors (Additional file 2: Figure S2e). Indeed, a positive correlation between *CD274* mRNA and miR-7702 was evident in both *TP53* genotypes (Additional file 2: Figure S2f).

### Members of immune-related pathways are enriched among the genes correlating best with *CD274* mRNA expression in TCGA dataset

**Fig. 2:**
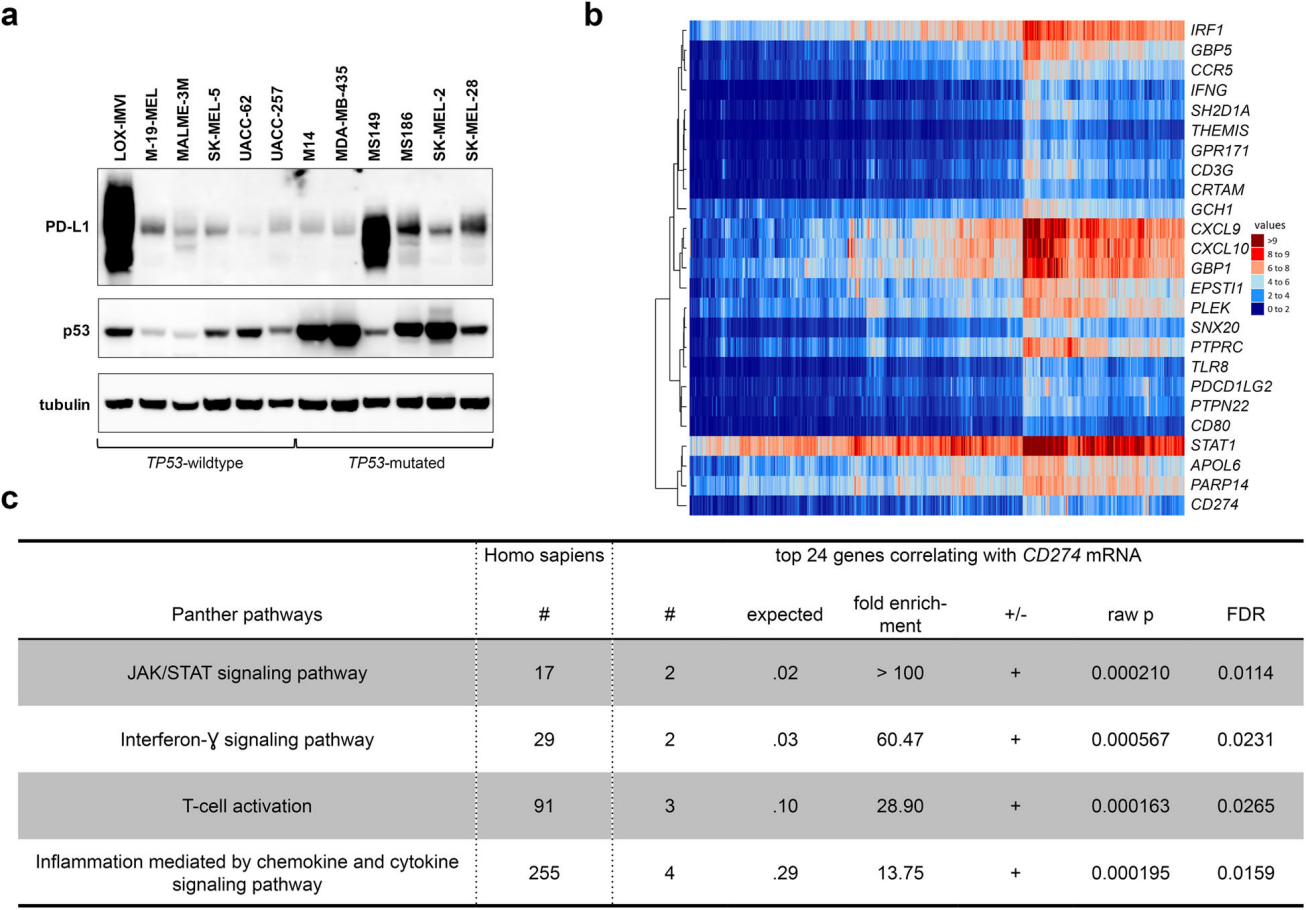
*CD274* expression correlates with genes of immune-related pathways while PD-L1 levels lack correlation with p53’s transcriptional activity. **a** Immunoblot for PD-L1 and p53 expression of each six untreated *TP53-*wildtype and -mutated melanoma cell lines. Before lysis, cells were cultured for at least three days. Blot is representative of two individual experiments. **b** Correlation of *CD274* with other genes was calculated using Spearman correlation (*n* = 447). Only genes with > 1 TPM in a least 20% of cases were included. The 24 best correlating genes are presented in a “heatmap”. Expression values are presented in a spectrum of blue (small) to red (high). **c** Gene enrichment analysis of the 24 best correlating genes with PANTHER overrepresentation test and Fisher test statistics depicting overrepresented pathways. Total number (#) of genes ascribed to the respective pathway, number of genes from the top 24 genes belonging to the pathway, expected frequency, fold enrichment, overpresentation indicated by “+”, raw *p*-value and false discovery rate (FDR) are given. *p* < 0.05 is regarded as statistically significant. TPM, transcripts per million

So far, our results revealed an increased *CD274* mRNA expression in the TCGA data and a higher percentage of PD-L1 positivity in *TP53*-mutated tumors in our cohort of 81 samples, while overall PD-L1 expression levels were not significantly different. We thus analyzed 6 *TP53-*wt and 6 *TP53-*mutated melanoma cell lines for PD-L1 expression. Our immunoblot analysis revealed that - as expected [47] - p53 expression levels were mostly higher in mutated cell lines, but no consistent difference in basal PD-L1 expression was evident when stratified by *TP53* status (Fig. 2a).

Hence, to identify factors, which modulate *CD274* mRNA expression, we searched for the 24 genes with the best correlation to *CD274* mRNA expression (Fig. 2b). In order to determine pathways that were overrepresented among these 24 genes, we analyzed them with PANTHER [45, 46]. This analysis demonstrated that the only pathways overrepresented by the genes are immune response-related (Fig. 2c), indicating that an ongoing immune response with IFN-ɣ secretion and activation of the JAK/STAT pathway might impact PD-L1 expression.

These observations prompted us to perform in vitro experiments in order to determine the role of p53 in the regulation of PD-L1 expression.

### p53 knockdown increases basal, but negatively affects IFN-ɣ-induced PD-L1 expression in melanoma cells

**Fig. 3:**
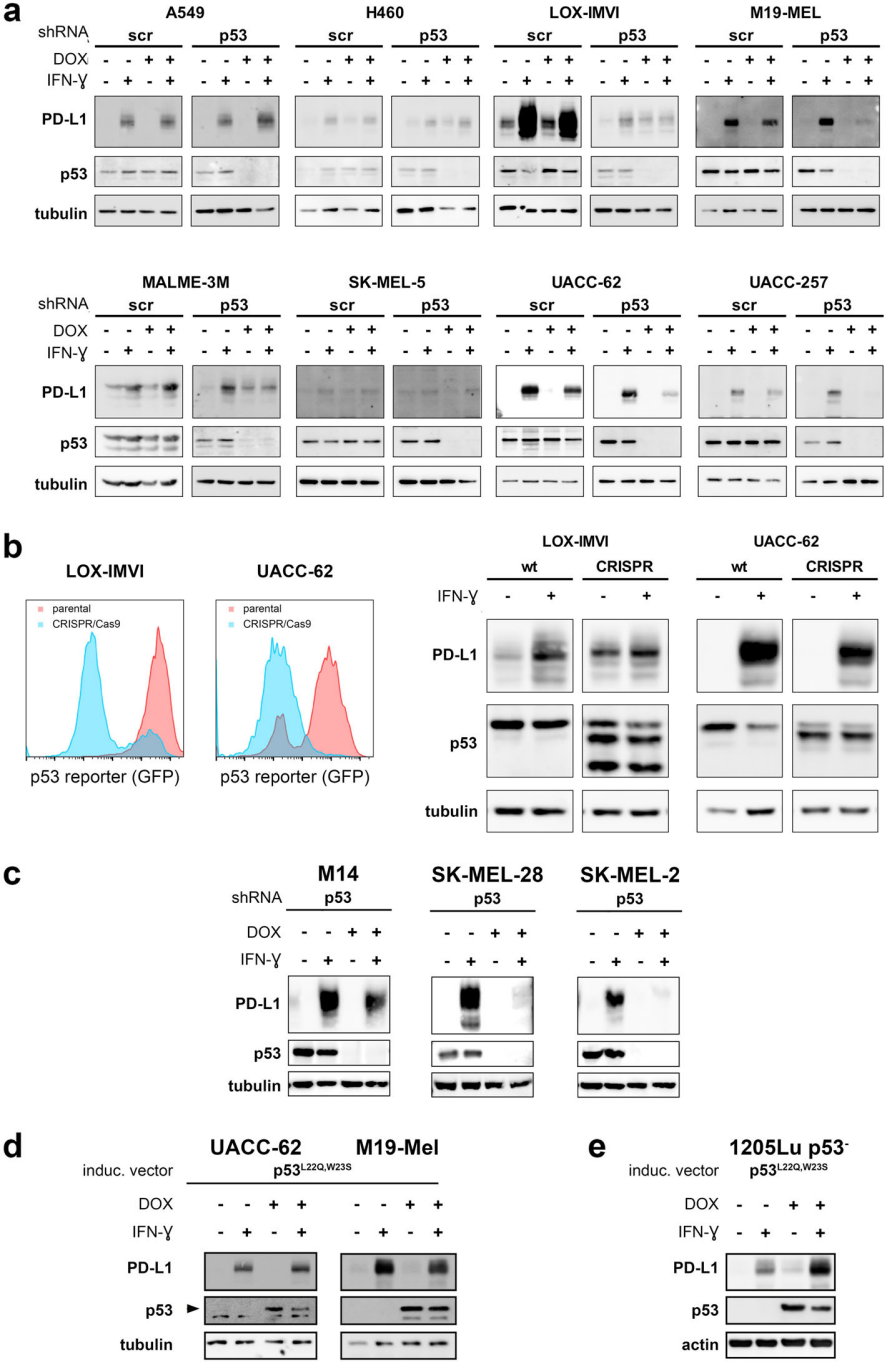
Presence of p53 protein, but not its transcriptional activity determines extent of IFN-ɣ induced PD-L1 expression in melanoma. **a** Immunoblot for PD-L1 and p53. NSCLC cell lines A549 and H460 served as control; all other are melanoma cell lines. IFN-ɣ treatment was for 48 h. **b** p53 was rendered transcriptionally inactive by introducing deletions using CRISPR/Cas9 technology and gRNA targeting exons 7 and 9 (LOX-IMVI) or exons 8 and 9 (UACC-62), respectively. Loss of transcriptional activity was determined by expression of GFP-based p53 reporter (left histograms; red: parental cells; blue: cells after CRISPR/Cas9 genome editing). Protein expression of p53 and PD-L1 in the absence or presence of IFN-ɣ for 48 h was determined by immunoblot. **c** Immunoblot for PD-L1 and p53 in *TP53*-mutated melanoma cells upon shRNA-mediated p53 knockdown. Cells were treated with IFN-ɣ as their *TP53*-wildtyp counterparts described in (**a**). p53 knockdown was achieved by culturing cells in doxycycline for 6 days. **d**, **e** Two *TP53*-wt melanoma cell lines (**d**) or a *TP53*-knockout cell line (**e**) were transduced with doxycycline-inducible p53^L22Q,W23S^ expression construct. Cells were incubated with doxycycline and treated with IFN-ɣ for 48 h, as described before. Expression of indicated proteins was determined by immunoblot. Arrow (**d**) indicates ectopic p53 expression. Please note, that for M19-MEL (**d**) the ectopic p53 expression was so much stronger than the endogenous that, in those samples without doxycycline, the signal for endogenous p53 was too low to be detected. ß-tubulin (**a**-**d**) or actin (**e**) served as loading controls. DOX, doxycycline. All blots are representative of two individual experiments

In order to directly test the impact of p53 on PD-L1 expression, we generated several cell lines with inducible shRNA targeting *TP53*. Two *TP53-*wt NSCLC cell lines, i.e. A549 and H460, which in another study demonstrated increased PD-L1 expression upon p53 knockdown or miR-34a transfection, served as positive controls [27]. Indeed, H460 cells displayed a slightly increased expression of PD-L1 upon p53 knockdown while expression levels were unaffected in scr control cells (Fig. 3a). Among the 6 p53 wt melanoma cells tested (LOX-IMVI, M19-MEL, MALME-3 M, SK-MEL-5, UACC-62, UACC-257), a modest increase of PD-L1 upon p53 knockdown was only evident for LOX-IMVI and MALME-3 M. For SK-MEL-5 there was no difference, and because for all others melanoma lines basal PD-L1 expression levels were below the detection limit of the immunoblot, we analyzed PD-L1 expression also by flow cytometry. This analysis demonstrated that in all analyzed cell lines a slight increase of PD-L1 expression was measurable upon p53 knockdown which, however, was not significant in any cell line (Additional file 3: Figure S3a).

It is known from the literature as well as apparent from our TCGA data analysis that PD-L1 expression is modified by immune responses leading to IFN-ɣ secretion [11, 12]. We therefore analyzed the effect of p53 knockdown on IFN-ɣ-induced PD-L1 expression. To this end, after culture for 4 days in the absence or presence of doxycycline to induce knockdown of p53, cells were additionally treated for 48 h with IFN-ɣ, and PD-L1 expression was determined by immunoblot or flow cytometry. As expected, IFN-ɣ did increase PD-L1 expression in all cell lines (Fig. 3a, Additional file 3: Figure S3a). This increase was much more pronounced than the increases observed upon p53 knockdown. Only for SK-MEL-5 the increase upon IFN-ɣ treatment was marginal. In the two NSCLC cell lines, IFN-ɣ-induced PD-L1 upregulation was similar or slightly increased upon additional p53 knockdown (Fig. 3a, Additional file 3: Figure S3a). Surprisingly, however, in the five melanoma cell lines (LOX-IMVI, M19-MEL, MALME-3 M, UACC-62, UACC-257) demonstrating a distinct IFN-ɣ-induced PD-L1 upregulation in our immunoblot analyses, a reduction of p53 protein levels was accompanied by a decreased IFN-ɣ-inducible PD-L1 expression. Indeed, the most prominent decrease of PD-L1-inducibilty was observed in those cell lines that presented with the strongest PD-L1 induction upon IFN-ɣ treatment (M19-MEL, UACC-62 and UACC-257, Fig. 3a, Additional file 3: Figure S3a).

Since shRNA might have off-target effects, we repeated these experiments in four melanoma cell lines with a second *TP53-*targeting shRNA. Although knockdown efficiency of this shRNA was not as good, we could still confirm our observation that upon p53 knockdown IFN-ɣ treatment was less effective in inducing PD-L1 expression (Additional file 3: Figure S3b).

Altogether, p53 knockdown in *TP53*-wildtype melanoma cells resulted in only a very modest increase of basal PD-L1 expression, but clearly reduced IFN-ɣ induced expression.

### The extent of IFN-ɣ-inducible PD-L1 expression is dependent on the presence of p53 protein, but not on its transcriptional activity

Our analyses of publicly available data had revealed that there is a positive correlation between PD-L1 and p53 expression. Further analyses demonstrated that *IFNG* mRNA levels were not different between tumors with *TP53*-wt or *TP53*-mutant status (Additional file 4: Figure S4a). Moreover, while *IFNG* levels did not correlate with p53 expression, there was a positive correlation with PD-L1 expression for both *TP53* genotypes (Additional file 4: Figure S4b, c). Taken together, these observations suggest that while p53 presence augments IFN-ɣ-induced PD-L1 expression, this might not dependent on its transcriptional activity. In order to test this hypothesis, we rendered p53 transcriptionally inactive in originally *TP53*-wt melanoma cells by introducing deletions in the DBD/HD using CRISPR/Cas9 technology. These two generated melanoma cell lines expressed truncated p53 which resulted in diminished p53 reporter activity. The extent of PD-L1 induction following IFN-ɣ treatment was, however, not affected (Fig. 3b).

Furthermore, we analyzed whether the effect observed for p53 knockdown in the wildtype *TP53* melanoma cell lines could be reproduced in three *TP53*-mutant cell lines (M14, SK-MEL-2, SK-MEL-28). Similar to *TP53-*wt melanoma cell lines, the reduction of p53 protein in these cells led to an impaired induction of PD-L1 by IFN-ɣ (Fig. 3c).

The ability to activate gene transcription is among the best-characterized properties of p53. This function is ascribed to the two transactivation domains (TADs), and introducing mutations at the amino acids 22 and 23 generates a p53 protein (p53^L22Q,W23S^) with clearly reduced transactivation-potential [34]. Hence, to further evaluate the role of transcriptional activity for IFN-ɣ-induced PD-L1 expression, we transduced *TP53*-wt melanoma cell lines or a *TP53*-knockout cell line with a vector allowing inducible expression of p53^L22Q,W23S^. When expressed in *TP53*-wt melanoma cell lines, PD-L1 inducibility by IFN-ɣ was hardly affected (Fig. 3d). Importantly, however, expression in a *TP53-*knockout melanoma cell line was already associated with an increased basal PD-L1 expression, and boosted PD-L1 expression upon IFN-ɣ stimulation (Fig. 3e). Thus, even when transactivation activity is impaired, the presence of p53 can augment IFN-ɣ-induced PD-L1 expression.

### p53 knockdown leads to a reduction of JAK2, which is associated with a delayed JAK2 and a diminished STAT1 phosphorylation by IFN-ɣ

**Fig. 4:**
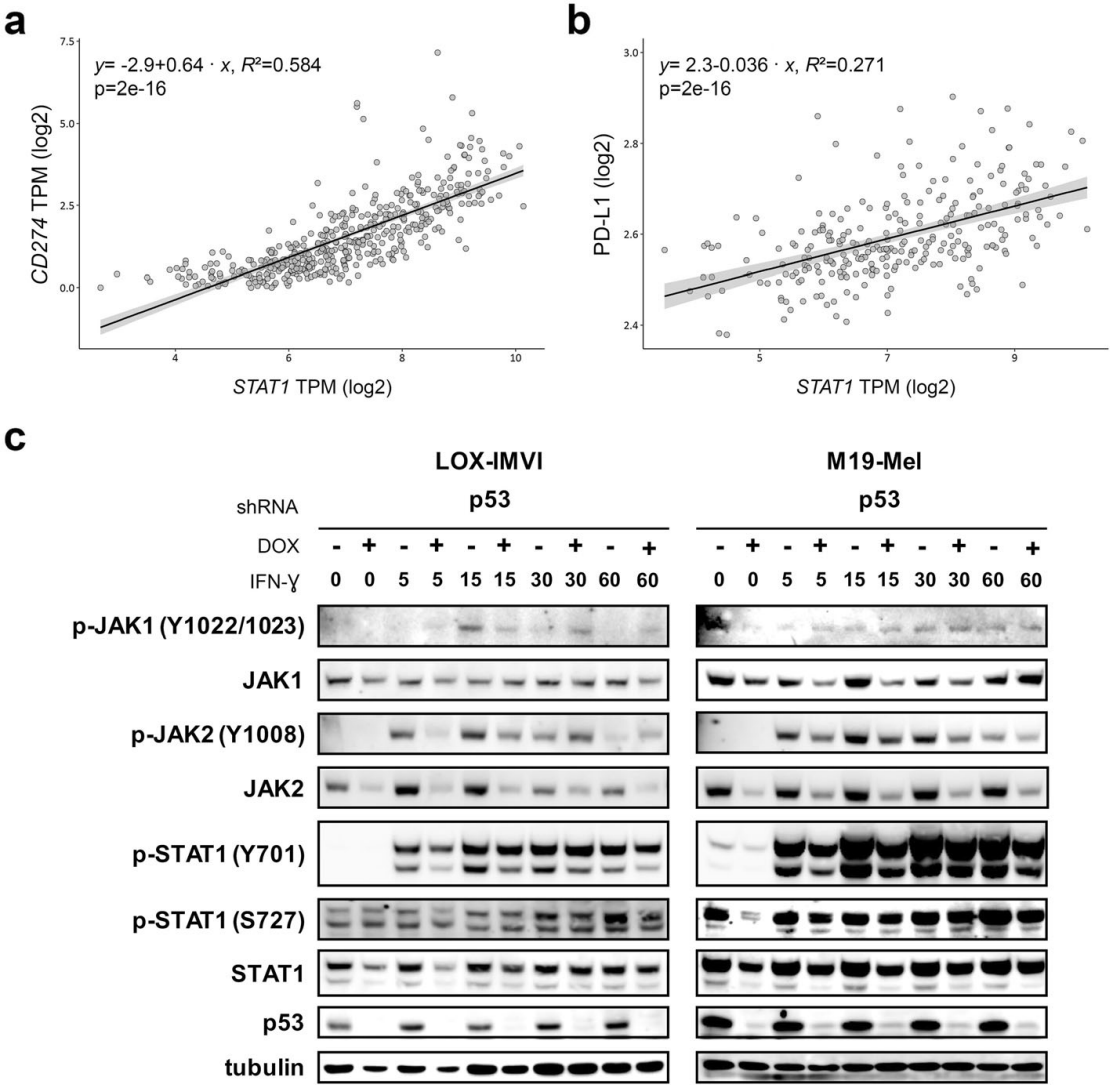
Correlation of *STAT1* and *CD274*/PD-L1 in melanoma, and disturbed IFN-ɣ signaling upon p53 knockdown in melanoma cell lines. **a**, **b** Linear regression analyses of *STAT1* mRNA with *CD274* mRNA (*n* = 347) (**a**) or PD-L1 protein (*n* = 262) (**b**). **c** LOX-IMVI and M19-MEL were incubated in six well plates for 6 days with doxycycline for p53 knockdown and subsequently treated with IFN-ɣ for 5, 15, 30 or 60 min. Effect of treatment on the IFN-ɣ signaling pathway was analyzed by immunoblot with the indicated antibodies. ß-tubulin served as a loading control. *p* < 0.05 is regarded as statistically significant. DOX, doxycycline; TPM, transcripts per million

**Fig. 5:**
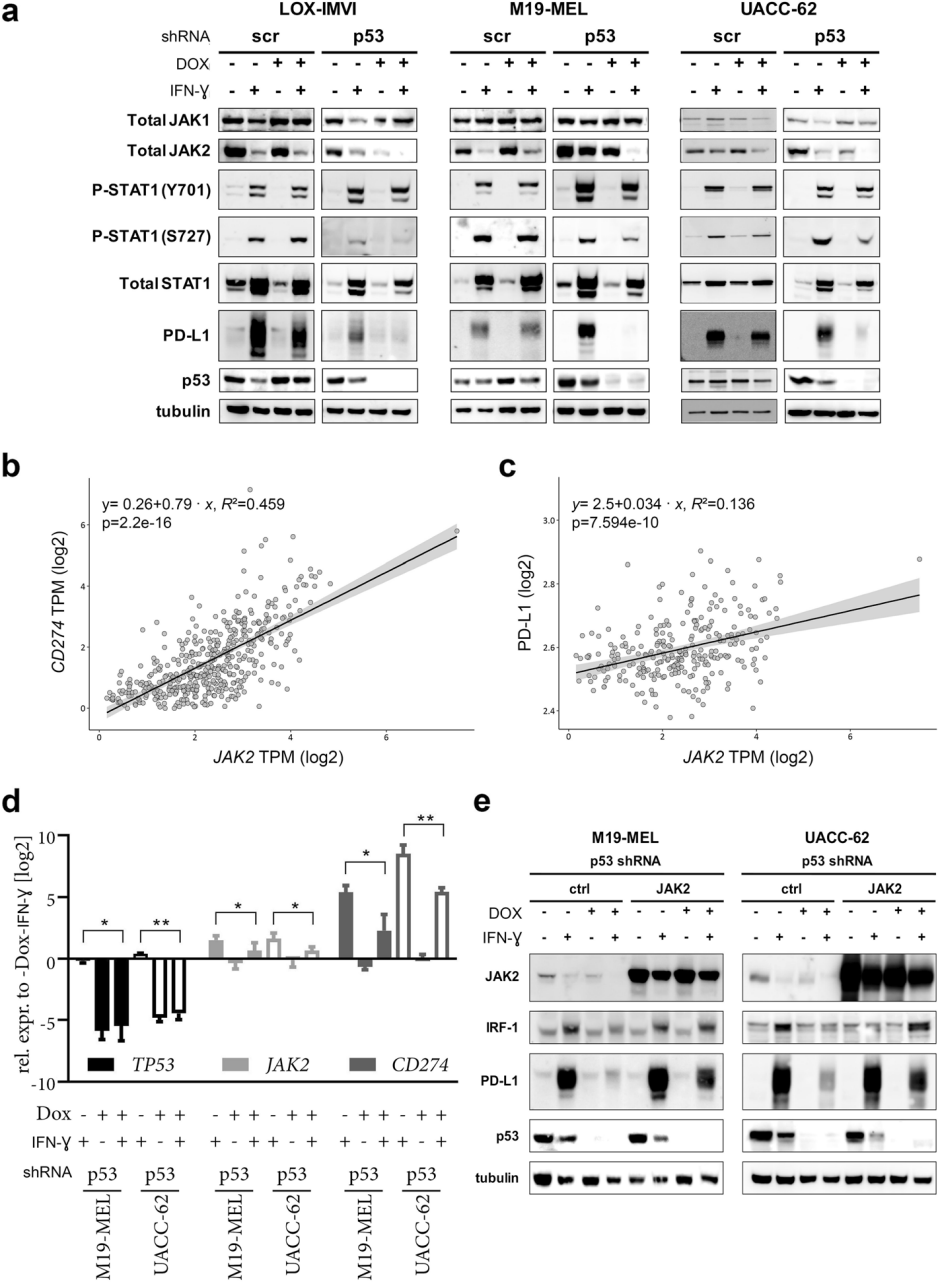
Restoration of p53 knockdown-associated JAK2 downregulation improves IFN-ɣ-inducible PD-L1 expression. **a** Immunoblot of three melanoma cell lines containing either an inducible *TP53*-targeting or a scr shRNA expression vector. IFN-ɣ treatment was for 48 h. **b**, **c** Linear regression analyses of *JAK2* mRNA with *CD274* mRNA (**b**; *n* = 347) or PD-L1 protein (**c**; *n* = 262). **d** M19-MEL and UACC-62 cells containing an inducible *TP53*-targeting shRNA vector were treated for 6 days with doxycycline with the last 2 days either in absence or presence of IFN-ɣ. After RNA isolation and cDNA generation, real-time quantitative PCR was performed for determination of *TP53*, *JAK2* and *CD274* mRNA expression. Relative expressions were calculated by the ΔΔCq method to the respective cell line sample without doxycycline and IFN-ɣ treatment. After log2 transformation, the means + SD of three independent experiments are depicted. Significant differences are indicated by stars (* < 0.05; ** < 0.01; paired t-test). **e** Two melanoma cell lines containing Doxycycline-inducible p53 shRNA were transduced with a JAK2 expression construct. Control cells and JAK2-overexpressing cells were incubated with doxycycline and treated with IFN-ɣ for 48 h as described before. Expression of indicated proteins was determined by immunoblot. ß-tubulin served as a loading control. *p* < 0.05 is regarded as statistically significant. DOX, doxycycline; scr, scramble; TPM, transcripts per million, ctrl, control. All blots are representative of two individual experiments

As mentioned before, IFN-ɣ signals through the JAK-STAT-IRF1 axis to regulate PD-L1 [18]. Notably, among the top 24 genes whose mRNA correlated with *CD274* mRNA in the TCGA SKCM data set, the best correlation was for *STAT1* (*p* = 2 × 10^− 16^; *R*^*2*^ = 0.584, Figs. 2c, 4a), which translated also in a positive correlation of *STAT1* mRNA and PD-L1 (*p* = 2 × 10^− 16^; *R*^*2*^ = 0.271, Fig. 4b). Importantly, STAT1 activation by genotoxic agents has been demonstrated to depend on p53 protein but not on its transcriptional activity [48]. In this regard, STAT1 possesses two phosphorylation sites (Y701 and S727), both of which are functionally important for efficient signaling through the type II interferon receptor pathway [19]. STAT1 Y701 phosphorylation depends directly on activated JAK1/2, whereas STAT1 S727 phosphorylation is fundamental for maximal ability to activate transcription of target genes, and can be modulated by different cellular influences [19, 49]. We first examined the effect of short term IFN-ɣ treatment, i.e. after 5, 15, 30 or 60 min, on the JAK/STAT pathway in two melanoma cell lines. These analyses revealed that total JAK2 and to a lesser extent STAT1 were reduced in p53 knockdown cells. Shortly after addition of IFN-ɣ, JAK2 became phosphorylated at Y1008 and STAT1 at Y701 while the phosphorylation of STAT1 at S727 was unchanged. In cells with reduced p53 levels, absolute JAK2 Y1008 phosphorylation levels were at least at 5 and 15 min lower; although the ratio of phosphorylated to total JAK2 was even higher due to decreased total JAK2 levels. Similarly, although more of STAT1 is phosphorylated at Y701, the total amount is slightly reduced due to accompanied decreased total-STAT1 levels (Fig. 4c). Of note, treatment of cells with IFN-ɣ for 48 h generally led to a decrease of JAK2 in melanoma cells, which was even more evident in cells with p53 knockdown (Fig. 5a). At this time point, STAT1 S727 phosphorylation was reduced in p53 knockdown cells.

### Ectopic JAK2 can largely restore reduced IFN-ɣ-inducible PD-L1 expression after p53 knockdown

Besides a reduced JAK2 expression upon p53 knockdown, we also detected within the TCGA-SKCM data a positive correlation between *CD274* mRNA and *JAK2* mRNA (*p* = 2.2 × 10^− 16^; *R*^*2*^ = 0.459) as well as between PD-L1 and *JAK2* mRNA (*p* = 7.6 × 10^− 10^; *R*^*2*^ = 0.136; Figs. 5b and c). To test, if reduction of IFN-ɣ-induced JAK2 and PD-L1 protein levels after p53 knockdown is accompanied with a decrease of corresponding mRNAs, we performed real-time PCR. These analyses revealed that p53 knockdown had no dramatic impact on basal *JAK2* or *CD274* mRNA expression levels in the 2 melanoma cell lines M19-MEL and UACC-62. Upon stimulation with IFN-ɣ, cells demonstrated enhanced *JAK2* and *CD274* mRNA expression. When combined with p53 knockdown, these increases were significantly reduced (Fig. 5d).

Based on our observations, we hypothesized that reduced JAK2 levels upon p53 knockdown are at least partially responsible for the decreased IFN-ɣ-induced PD-L1 expression. Therefore, we analyzed p53 knockdown in two melanoma cell lines ectopically expressing JAK2. In those cells, JAK2 levels were higher than endogenous levels in control cells. While IFN-ɣ-induced expression of interferon regulatory factor 1 (IRF-1) was reduced by p53 downregulation in control cells, ectopic expression of JAK2 led to levels similar to that in control cells without p53 downregulation. Importantly, JAK2 overexpression largely restored IFN-ɣ-induced PD-L1 expression in p53 knockdown cells (Fig. 5e).

## Discussion

p53 is a central tumor suppressor protein, which is stabilized and activated following different cellular stresses including DNA damage and replication stress provoked by deregulated oncogenes [29]. Once activated this transcription factor can promote cell cycle arrest, DNA repair or apoptosis. Importantly, the specific p53 triggered response depends on the cellular context, which includes cell type, epigenetic state, tissue microenvironment and activating signals [29, 50]. In particular, it has been proposed that melanocytes (and accordingly melanocyte-derived tumor cells) may respond differently to p53 activation because these cells are adopted to survive even with p53 induction by highly mutagenic UV light and by the oxidative stress of melanin production [51].

Besides the response to DNA damage, p53 controls many further distinct processes and plays e.g. an important role in inflammation and immune responses [52]. Indeed, p53 is directly involved in the upregulation of antigen presentation via the major histocompatibility complex (MHC) I pathway [53]. Reported mechanisms include induction of members of the antigen processing machinery like TAP1 or ERAP1 by wildtype p53 [54, 55]. As a result, wildtype p53 can improve MHC class I expression and thereby promote tumor cell killing by cytotoxic T-lymphocytes (CTL) [53–55]. Therefore, besides several other aspects of p53 biology, also immune modulation may contribute to the impact that *TP53* mutation status has on prognosis and even more on immunotherapy response. Indeed, *TP53* mutation has been demonstrated to be associated with poorer outcome in melanoma patients receiving anti-CTLA-4 treatment [56]. In another study with melanoma patients treated by different immune checkpoint blockade therapies, *TP53* mutation was one of the factors associated with inferior outcome [57]. In melanoma, the best known predictor for response to anti-PD-1 therapy is the expression of PD-L1 on tumor cells [3, 4, 6, 8–10]. In this regard, in NSCLC it has been demonstrated that p53 downregulates PD-L1 via miR-34a and thereby enhances CTL activity [27]. We thus analyzed publically available mRNA and protein expression data as well as own samples to investigate whether *TP53* might influence PD-L1 expression in melanoma. For *CD274* mRNA we obtained similar results to those described for NSCLC, with a higher expression level for *TP53*-mutated tumors and an inverse correlation between *TP53* and *CD274* mRNA for *TP53* wt melanoma tumors (Additional file 1: Figure S1b). This correlation, however, was quite weak, and the underlying mechanism of regulation appears to be different from that reported for NSCLC, since we could not observe a correlation for miR-34a and *CD274* mRNA indicating that specific p53 activity is context-dependent [29, 50]. Moreover, we detected a positive correlation of p53 and PD-L1 at the protein level. When stratified by *TP53* status, PD-L1 expression levels were not different although p53 expression was higher in *TP53*-mutated tumors (data not shown). Since RPPA protein expression data cannot distinguish between expression on tumor or stroma cells, we analyzed a series of melanoma samples with known *TP53* status by immunohistochemistry revealing that PD-L1 tumor cell positivity was more frequent in *TP53*-mutated tumors. This observation certainly has to be confirmed in a larger cohort. Nevertheless, this may match the situation of primary pulmonary lymphoepithelioma-like carcinoma, where immunohistochemically p53-positive samples, which - based on their staining pattern - were regarded as *TP53*-mutated, and were also significantly more often PD-L1-positive [58]. In contrast, the *TP53* mutation status did not correlate with PD-L1 expression in colon cancer, suggesting again diverging mechanisms of PD-L1 regulation in different tumor types [59]. Given that our immunoblot results did not reveal a clear correlation between *TP53*-mutation status and PD-L1 baseline expression in melanoma cell lines (Fig. 2a), and p53 knockdown only slightly influenced PD-L1 baseline expression, it is unlikely that p53 has a major intrinsic effect on PD-L1 expression in melanoma.

Accordingly, TCGA data analyses demonstrated that, among the mRNAs best correlating with *CD274* expression, only immune response-related genes were enriched. This indicates that an ongoing immune response may determine PD-L1 expression. Indeed, immunohistochemistry of many cancers revealed PD-L1 expression typically in T-cell–rich areas of tumors, particularly at the invasive margin, illustrating IFN-ɣ-inducible PD-L1 expression to be more common than constitutive expression [12, 60, 61].

This in vivo observation can be reenacted by exposing tumor cells to IFN-ɣ, thereby leading to a marked increase of PD-L1 expression (Knol et al. [62] and Fig. 3a). Surprisingly, this increase was diminished upon p53 knockdown in melanoma cells. Importantly, decreased IFN-ɣ-induced PD-L1 expression upon p53 knockdown was also evident in *TP53-*mutated melanoma cell lines, while rendering p53 inactive by CRISPR/Cas9 had no effect on PD-L1 inducibility. Furthermore, a histopathological study of desmoplastic melanoma, which frequently harbor *TP53* mutations, identified a significant positive correlation between PD-L1 and p53 expression [63, 64]. Hence, presence of p53 but not its transcriptional activity appears to be required for full IFN-ɣ-induced PD-L1 expression in melanoma. Consistently, expression of p53^L22Q,W23S^, a p53 protein with impaired transactivation activity, in the 1205Lu *TP53*-knockout^−^ melanoma cell line led to a prominent increase of IFN-ɣ-induced PD-L1 expression. In this context, it certainly would also be interesting to analyze the impact of p53 with GOF mutations on IFN-ɣ-induced PD-L1 expression, an aspect we did not yet address.

The many functions of p53 can be divided into transcription-dependent and -independent activities. Indeed, besides in the nucleus p53 can act also in the cytosol or at the mitochondria [65]. Regarding apoptosis promotion, it has been demonstrated that the pro-apoptotic protein Bax can be activated by certain transcription-deficient mutant p53 proteins [66]. As another example, genotoxic drugs can induce STAT1 activation, a process that depends on p53 protein but not on its transcriptional activity. This has been demonstrated by restoring drug-induced STAT1 Y701 phosphorylation by expression of transcriptional-inactive p53 mutants in p53-null cell lines [48]. Of note, it has previously been demonstrated that genotoxic stress-induced upregulation of PD-L1 is also p53-dependent in a breast carcinoma cell line [67].

In our experiments, reduced total-STAT1, but also STAT1 S727 phosphorylation was evident after 48 h of IFN-ɣ-stimulation in p53 knockdown cells. Importantly, phosphorylation of this STAT1 site is induced by various stimuli (e.g. LPS, PDGF) besides IFN-ɣ signaling, and is essential for maximal transcription of target genes [19, 49]. Therefore, decreased STAT1 S727 phosphorylation through molecules beyond JAK-STAT-signaling, could have contributed to the diminished IFN-ɣ induced PD-L1 expression after p53 knockdown.

Furthermore, it has been shown that inducible but not constitutive PD-L1 expression depends on NF-κB activation in melanoma cells [68]. Interestingly, while NF-κB and p53 often have opposing effects in cancer cells, in human monocytes and macrophages both co-regulate induction of pro-inflammatory genes [69].

Our real-time PCR results indicate that IFN-ɣ-induced upregulation of JAK2 and PD-L1 is already affected at mRNA level (Fig. 5d). This sustains our hypothesis that p53 knockdown impairs IFN-ɣ-induced *CD274* transcription mainly through interference with the JAK-STAT signaling pathway. Consistently, we revealed that p53 knockdown was associated with a reduction of JAK2 protein levels (Figs. 4c, 5a and d). Luo et al. recently demonstrated that JAK2 knockdown in various melanoma cell lines only diminished PD-L1, but did not substantially change IFN-ɣ-induced MHC-I expression [70]. In contrast, in an NSCLC cancer cell line p53 cooperated with IFN-ɣ to enhance the expression of surface MHC-I [54]. Naturally, we also analyzed IFN-ɣ induced MHC-I surface expression upon p53 knockdown and noted that induction was only slightly affected (Additional file 3: Figure S3c). This observation is in accordance with the results of Luo et al. and again indicates differences between melanoma and NSCLC [70].

## Conclusions

In summary, there is a tendency of higher PD-L1 expression in *TP53*-mutated melanoma cells. One contributing factor might be an increased p53 expression level in these tumors. Indeed, while our analyses demonstrate that p53 has only a minor influence on constitutive PD-L1 expression, its presence is important for IFN-ɣ-induced PD-L1 expression through the JAK-STAT-signaling pathway in melanoma cell lines. Moreover, it does not seem to be important whether p53 is transcriptionally active. Interestingly, short-term JAK2 inhibition in a preclinical melanoma model did not affect immunotherapeutic responses, while for melanoma patients inactivating JAK2 mutations have already been associated with a diminished response to anti-PD-1 directed immunotherapy [70–72]. Accordingly, future studies should analyze, whether p53 expression levels correlate with JAK2 expression in melanoma, and how they affect response to anti-PD-1-based immunotherapy.

## Supplementary information

**Additional file 1: Figure S1.** Clinical characteristics of patients included in immunohistochemistry study and weak negative correlation of *TP53* and *CD274* in *TP53*-wildtype TCGA samples. **(a)** A total of 81 samples were included in this analysis. ALM indicates acral lentiginous melanoma; LMM, lentigo maligna melanoma, NM, nodular melanoma; SSM, superficial spreading melanoma; mut, mutated; wt, wildtype. **(b)** Linear regression analysis of *TP53* mRNA with *CD274* mRNA (*n* = 347) was conducted separately for *TP53*-wt and -mutant samples. (JPG 1844 kb)

**Additional file 2: Figure S2.** miR-34a is lower expressed in *TP53*-mutated tumors but does not correlate with PD-L1 protein in melanoma. (**a**) Wilcoxon-Mann-Whitney test was applied to compare miR34a expression between *TP53-*wt and -mutant samples. (**b**) Linear regression analysis was used to analyze the relation between miR34a and PD-L1 protein. (**c**) Correlation of *CD274* with miRs was calculated using spearman correlation. Only miRs with > 1 RPM in a least 20% of cases were included. The 24 best correlating miRs are presented in a “heatmap”. Expression values are presented in a spectrum of blue (small values) to red (high values). (**d**) Linear regression analysis of miR7702 and *CD274*. (**e**) Wilcoxon-Mann-Whitney test was applied to compare miR7702 expression between *TP53-*wt and -mutant samples. (**f**) Linear regression analysis of miR7702 with *CD274* was conducted separately for *TP53*-wt and -mutant samples. *p* < 0.05 is regarded as statistically significant. Mut, mutated; TPM, transcripts per millions; RPM, reads per million; wt, wildtype. (JPG 1937 kb)

**Additional file 3: Figure S3.** IFN-ɣ induced PD-L1 expression is reduced after p53 knockdown in melanoma while HLA expression is hardly affected. (**a**) Flow cytometry for PD-L1 expression. Presented is the PD-L1 mean normalized to the mean of control cells cultured without DOX or IFN-ɣ for 48 h. The data are presented as the mean + s.e.m. of three independent experiments. *p < 0.05, ns = not significant. (**b**) The effect of a second *TP53*-targeting shRNA on IFN-ɣ-induced PD-L1 expression. Those melanoma cell lines, which had revealed the most distinctive reduction of IFN-ɣ-induced PD-L1 expression upon p53 knockdown by the first shRNA, were transduced by another inducible shRNA. IFN-ɣ treatment was for 48 h. PD-L1 and p53 expression was measured by immunoblot. ß-tubulin served as a loading control. Blot is representative of two individual experiments. (**c**) LOX-IMVI and UACC-257, transduced with the first inducible *TP53*-targeting shRNA, were treated with IFN-ɣ for 48 h. MHC-I (grey) and PD-L1 (black) expression was measured by flow cytometry. Depicted is the fluorescence mean normalized to the mean of control cells treated neither with doxycycline nor IFN-ɣ. The data is presented as the mean + s.e.m. of three independent experiments. *p < 0.05, **p < 0.01, ns = not significant. p53 knockdown was achieved by culturing cells in doxycycline for 6 days in all experiments. (JPG 1296 kb)

**Additional file 4: Figure S4.** No differential expression of *IFN-ɣ* between *TP53*-genotypes, but positive correlation of *IFN-ɣ* and PD-L1 expression for both genotypes. **(a)** Wilcoxon-Mann-Whitney test was applied to compare *IFN-ɣ* expression between *TP53-*wt and -mutant samples. **(b, c)** Linear regression analysis of *IFN-ɣ* with p53 protein (**b**) or with PD-L1 protein (**c**) was conducted separately for *TP53*-wt and -mutant samples. *p* < 0.05 is regarded as statistically significant. Mut, mutated; TPM, transcripts per millions; wt, wildtype. (JPG 591 kb)

## Data Availability

The data presented is partially obtained by analyzing already publicly available data. All other original data generated or analyzed during this study are included in the manuscript or can be accessed through its supplementary files.

## Abbreviations

*CD274*: gene coding for PD-L1 protein
Dox: Doxycycline
mut: mutated
RPM: Reads per million
*TP53*: gene coding for p53 protein
TPM: Transcripts per millions
wt: wildtype
