# Supplementary figures and images for: IFN-gamma-induced PD-L1 expression in melanoma depends on p53 expression
Source: J Exp Clin Cancer Res. 2019 Sep 11;38:397. doi: 10.1186/s13046-019-1403-9 (PMC6737652; doi:10.1186/s13046-019-1403-9)

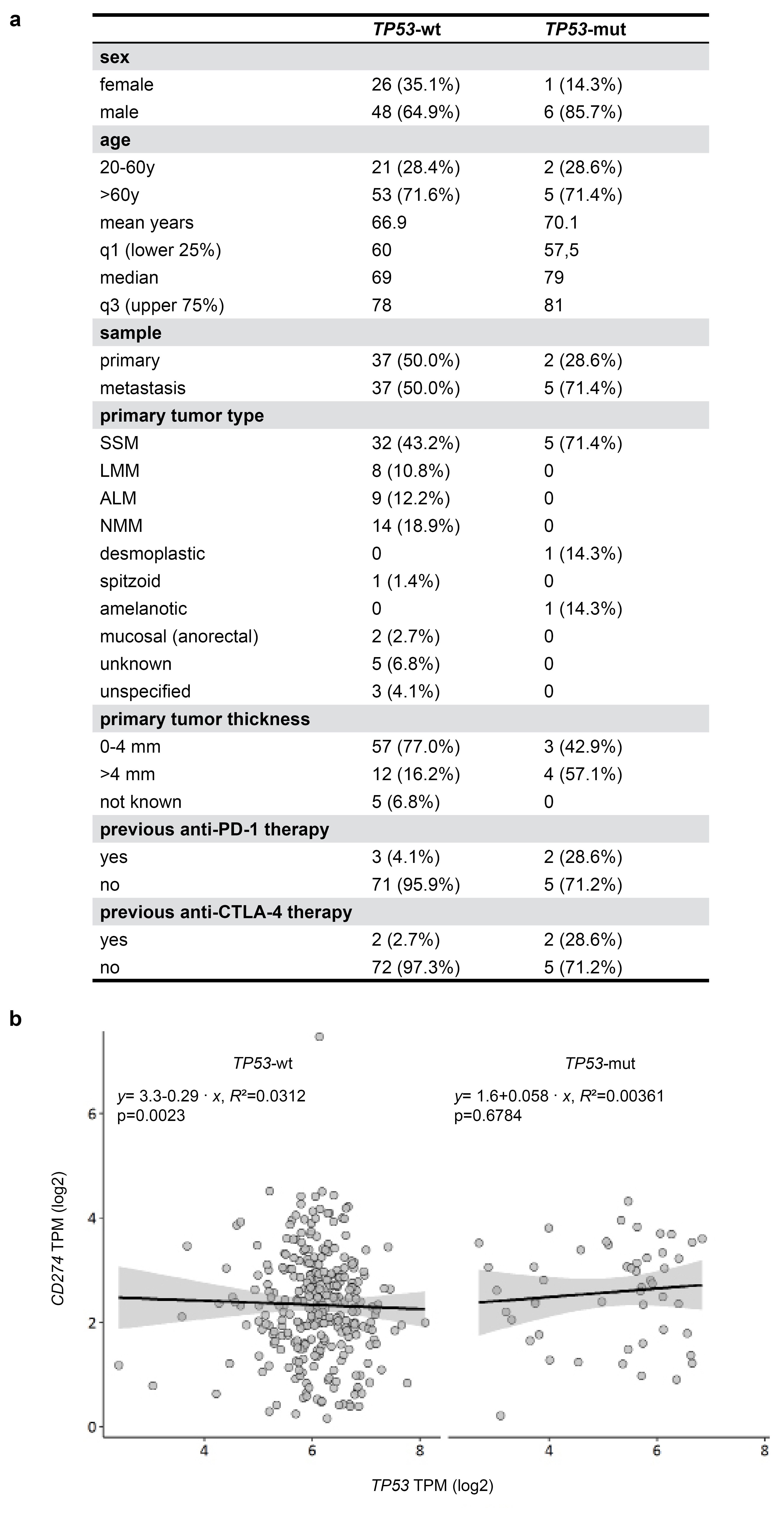

Supplement: Supplementary file 1 — Additional file 1: Figure S1. Clinical characteristics of patients included in immunohistochemistry study and weak negative correlation of TP53 and CD274 in TP53-wildtype TCGA samples. (a) A total of 81 samples were included in this analysis. ALM indicates acral lentiginous melanoma; LMM, lentigo maligna melanoma, NM, nodular melanoma; SSM, superficial spreading melanoma; mut, mutated; wt, wildtype. (b) Linear regression analysis of TP53 mRNA with CD274 mRNA (n = 347) was conducted separately for TP53-wt and -mutant samples. (JPG 1844 kb) [file 13046_2019_1403_MOESM1_ESM.jpg]

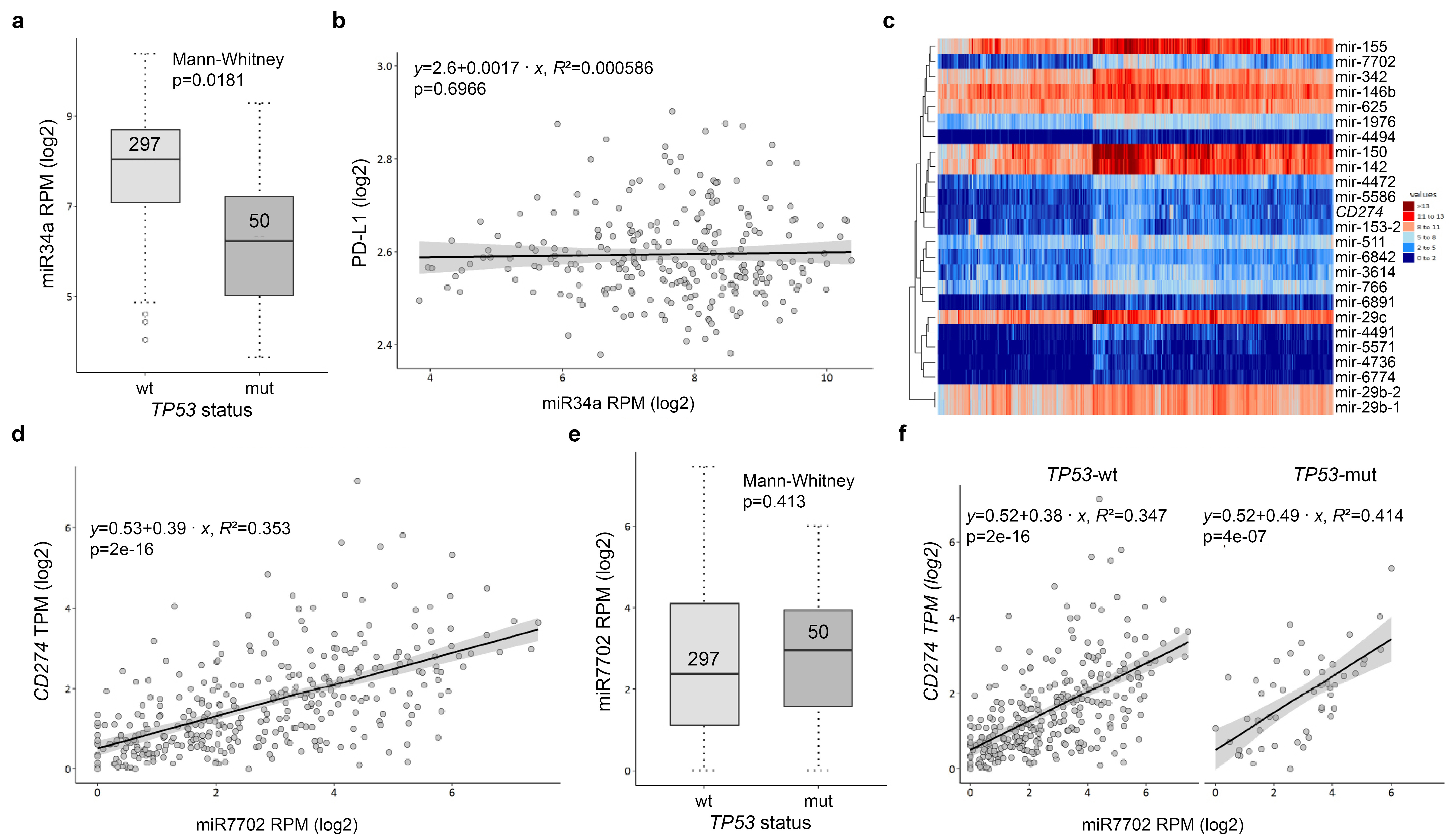

Supplement: Supplementary file 2 — Additional file 2: Figure S2. miR-34a is lower expressed in TP53-mutated tumors but does not correlate with PD-L1 protein in melanoma. (a) Wilcoxon-Mann-Whitney test was applied to compare miR34a expression between TP53-wt and -mutant samples. (b) Linear regression analysis was used to analyze the relation between miR34a and PD-L1 protein. (c) Correlation of CD274 with miRs was calculated using spearman correlation. Only miRs with > 1 RPM in a least 20% of cases were included. The 24 best correlating miRs are presented in a “heatmap”. Expression values are presented in a spectrum of blue (small values) to red (high values). (d) Linear regression analysis of miR7702 and CD274. (e) Wilcoxon-Mann-Whitney test was applied to compare miR7702 expression between TP53-wt and -mutant samples. (f) Linear regression analysis of miR7702 with CD274 was conducted separately for TP53-wt and -mutant samples. p < 0.05 is regarded as statistically significant. Mut, mutated; TPM, transcripts per millions; RPM, reads per million; wt, wildtype. (JPG 1937 kb) [file 13046_2019_1403_MOESM2_ESM.jpg]

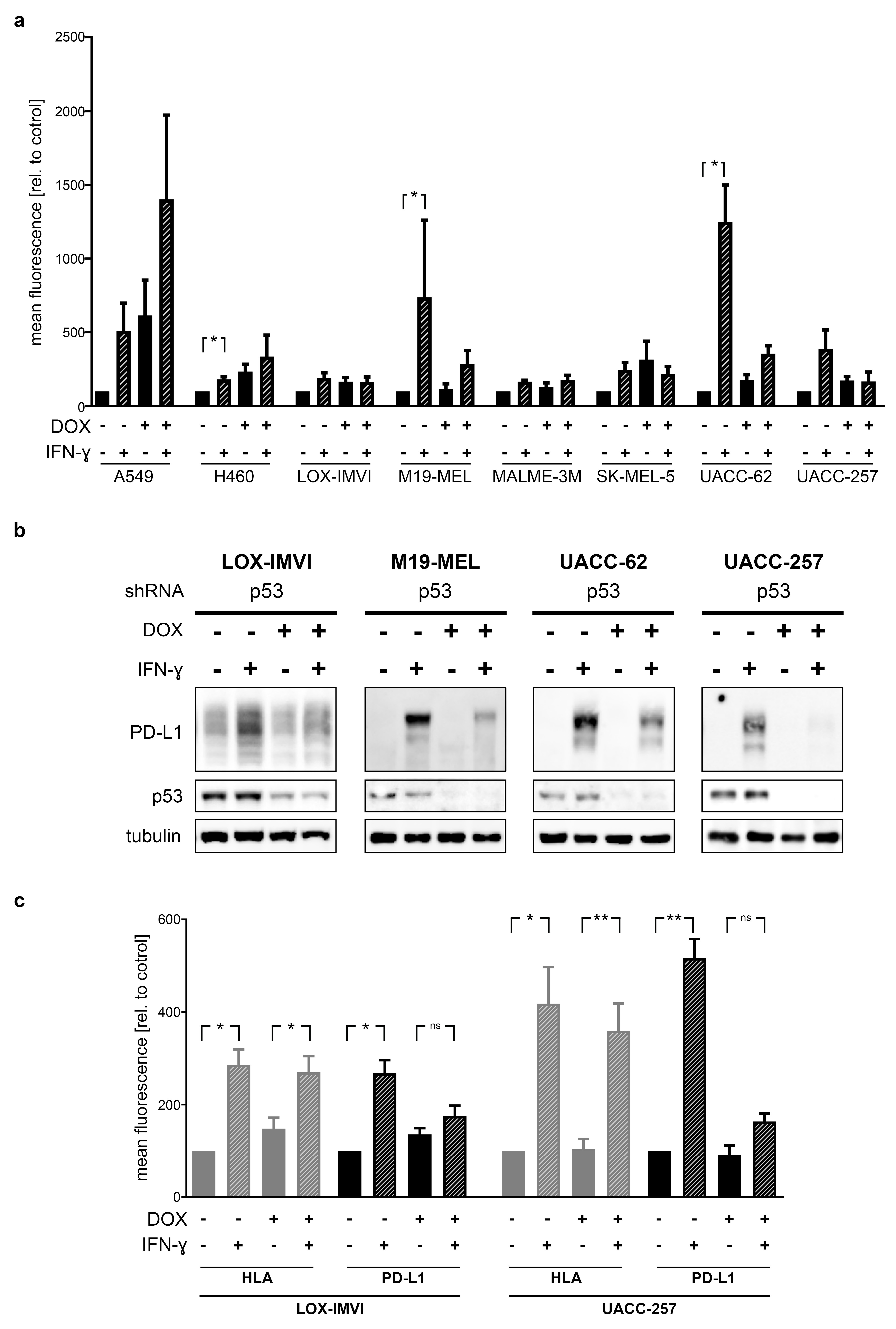

Supplement: Supplementary file 3 — Additional file 3: Figure S3. IFN-ɣ induced PD-L1 expression is reduced after p53 knockdown in melanoma while HLA expression is hardly affected. (a) Flow cytometry for PD-L1 expression. Presented is the PD-L1 mean normalized to the mean of control cells cultured without DOX or IFN-ɣ for 48 h. The data are presented as the mean + s.e.m. of three independent experiments. *p < 0.05, ns = not significant. (b) The effect of a second TP53-targeting shRNA on IFN-ɣ-induced PD-L1 expression. Those melanoma cell lines, which had revealed the most distinctive reduction of IFN-ɣ-induced PD-L1 expression upon p53 knockdown by the first shRNA, were transduced by another inducible shRNA. IFN-ɣ treatment was for 48 h. PD-L1 and p53 expression was measured by immunoblot. ß-tubulin served as a loading control. Blot is representative of two individual experiments. (c) LOX-IMVI and UACC-257, transduced with the first inducible TP53-targeting shRNA, were treated with IFN-ɣ for 48 h. MHC-I (grey) and PD-L1 (black) expression was measured by flow cytometry. Depicted is the fluorescence mean normalized to the mean of control cells treated neither with doxycycline nor IFN-ɣ. The data is presented as the mean + s.e.m. of three independent experiments. *p < 0.05, **p < 0.01, ns = not significant. p53 knockdown was achieved by culturing cells in doxycycline for 6 days in all experiments. (JPG 1296 kb) [file 13046_2019_1403_MOESM3_ESM.jpg]

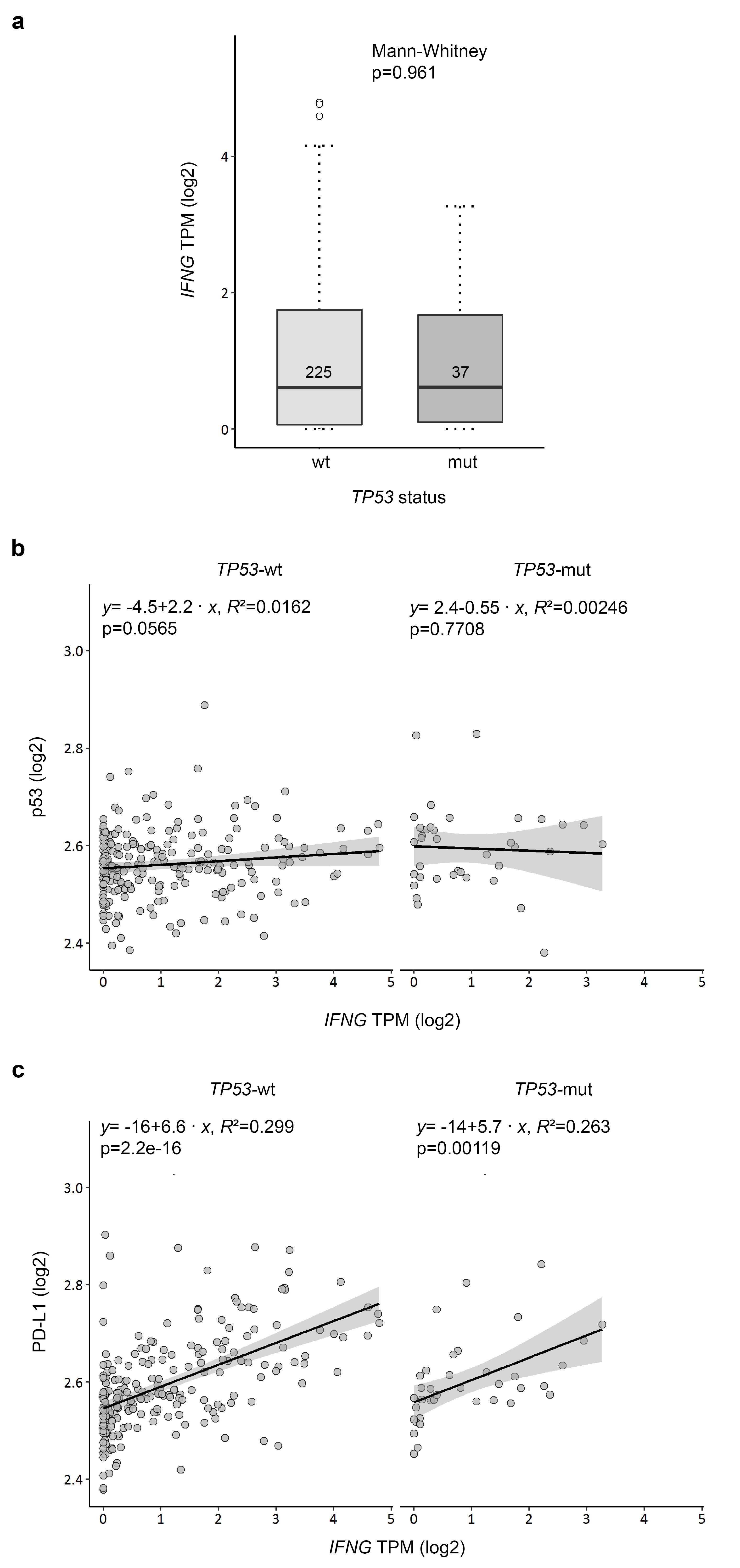

Supplement: Supplementary file 4 — Additional file 4: Figure S4. No differential expression of IFN-ɣ between TP53-genotypes, but positive correlation of IFN-ɣ and PD-L1 expression for both genotypes. (a) Wilcoxon-Mann-Whitney test was applied to compare IFN-ɣ expression between TP53-wt and -mutant samples. (b, c) Linear regression analysis of IFN-ɣ with p53 protein (b) or with PD-L1 protein (c) was conducted separately for TP53-wt and -mutant samples. p < 0.05 is regarded as statistically significant. Mut, mutated; TPM, transcripts per millions; wt, wildtype. (JPG 591 kb) [file 13046_2019_1403_MOESM4_ESM.jpg]
